# A scoping Review of Tools to Evaluate Existing Playgrounds for Inclusivity of Children with Disabilities

**DOI:** 10.3389/fresc.2023.1102490

**Published:** 2023-02-23

**Authors:** Leah G. Taylor, Mara Primucci, Leigh M. Vanderloo, Kelly P. Arbour-Nicitopoulos, Jennifer Leo, Jason Gilliland, Patricia Tucker

**Affiliations:** ^1^ Health and Rehabilitation Sciences, Western University, London, Ontario, Canada; ^2^ Child Health and Physical Activity Laboratory, Western University, London, Ontario, Canada; ^3^ School of Occupational Therapy, Western University, London, Ontario, Canada; ^4^ ParticipACTION, Toronto, Ontario, Canada; ^5^ Faculty of Kinesiology and Physical Education, Mental Health and Physical Activity Research Centre, University of Toronto, Toronto, Ontario, Canada; ^6^ Steadward Centre for Personal and Physical Achievement, Sport, and Recreation, University of Alberta, Edmonton, Alberta, Canada; ^7^ Department of Geography and Environment, Western University, London, Ontario, Canada; ^8^ Department of Paediatrics, Schulich School of Medicine, London, Ontario, Canada; ^9^ Department of Epidemiology and Biostatistics, Schulich School of Medicine, London, Ontario, Canada; ^10^ School of Health Studies, Western University, London, Ontario, Canada; ^11^ Children's Health Research Institute, Lawson Health Research Institute, London, Ontario, Canada

**Keywords:** playground, inclusion, child, disability, scoping review

## Abstract

**Introduction:**

Children with disabilities may be unable engage playground spaces due to barriers exacerbating exclusion. Therefore, clarity on how to evaluate existing playgrounds for inclusivity of children with disabilities is required.

**Methods:**

A scoping review was undertaken to explore auditing tools.

**Results:**

Fourteen white and grey literature resources were identified. The term “inclusion” was operationalized differently across tools, primarily focusing on physical accessibility. Characteristics of the tools were synthesized into 13 inclusive design recommendations for playgrounds. Two tools showed promise, evaluating 12/13 recommendations.

**Discussion:**

The results of this review provide guidance on existing tools for evaluating playgrounds for inclusion for community stakeholders and researchers.

**Systematic Review Registration:**

https://osf.io/rycmj.

## 1. Introduction

Play is a fundamental right of childhood (1), and has important implications for a child's wellbeing and healthy development (2). Children's environments shape opportunities for engaging in play, which further influences their physical health, social skills, and emotional wellbeing (3, 4). For example, access to community playgrounds (i.e., any fixed equipment used for play, typically found in parks, schoolyards, and childcare and recreation facilities: Canadian Standards Association) (5), is positively associated with children's levels of unstructured play (3), which supports increased physical activity levels and cardiorespiratory fitness, and decreased sedentary behavior (6–8). However, research suggests that children with disabilities experience exclusion in accessing and engaging in community playgrounds, and therefore, are less likely to reap the associated benefits (9, 10).

Children with disabilities can experience environmental barriers on playgrounds caused by inappropriate equipment (i.e., ground cover, inappropriate pathways, complex design, lack of alternative equipment, etc.), inadequate play options and play value, and limited opportunities for social interaction (11). These barriers impede full participation in play. This finding is concerning as globally, approximately 240 million children have a disability that limits their full participation in society (12). Creating engaging and inclusive environments, which support and facilitate children's participation, is crucial to extending the benefits of play to all children.

Inclusion emphasizes the full participation of individuals of all abilities by providing a space to interact, engage, and belong (13). Consequently, inclusive playground environments can facilitate entry to play and allow children with disabilities to feel like equal participants who can access and engage in the physical and social aspects of play (14). Inclusion encompasses design features such as accessibility (the physical ability of people to access the play space), useability (subjective perception of an individual's ability to engage in an activity within their environment), and playability (providing opportunities for individuals of all abilities to engage in play). Inclusion can be achieved through universal design; ensuring the built environment meets the needs of as many people, to the greatest extent possible (11).

It is important to consider how playground design can impact social inclusion and a child's right to play. In regards to inclusion and play-related policy, 196 countries have ratified the United Nations Convention on the Rights of the Child, which indicates that every child has the right to active participation in age-appropriate play, recreation, and their community (1). Furthermore, the United Nations Convention on the Rights of Persons with Disabilities has been ratified by 185 countries, and emphasizes that for individuals with disabilities to experience full inclusion in their communities, accessible, barrier-free physical and social environments are required (15).

On an international scale, some guidance is available *via* country-specific legislation governing new playground design (e.g., Australia/New Zealand: AS/NZS 4685, Australia: AS 4422; Britain and Europe: BS/EN1176; Canada: Z614:20; Brazil: NBR 16,071) (16). In 2010, the United States released the Americans with Disabilities Act Standards for Accessible Design (ADA Standards) (17). This resource provides minimum acceptable standards for designing accessible playgrounds relevant to American legislation, and are commonly referred to in global applications (16). In Canada, three out of 13 provinces/territories have enacted comprehensive accessibility laws (Ontario, Nova Scotia, and Manitoba); however, outside of Ontario, legislation governing accessibility on playgrounds is scarce (18).

While country-respective accessibility standards are a positive starting point for play opportunities for all children, they typically focus on recommendations for designing new, accessible playgrounds; this results in little guidance available for retrofitting established play structures. Furthermore, government policy alone is often insufficient to draw awareness to the specific barriers experienced by children using playgrounds in their communities (19). Research has indicated that playground accessibility and usability may not be successfully implemented despite accessibility standards being put in place (20). Clear guidance on tools available to evaluate existing structures is necessary to inform decision-making and address priorities (e.g., municipal funding, urban design, community health). This will aid in informing resource allocation for improving inclusivity for children with disabilities, to participate in play in their everyday environments.

Improved clarity on how to evaluate and retrofit or redesign existing playground structures for inclusivity is required. However, previous systematic and scoping reviews have not addressed how to evaluate existing playgrounds for inclusion, but instead focus on best practices for designing new structures (21–24). While applying design best practices for new playgrounds is a positive starting point to examine inclusion on existing playgrounds, auditing tools should quantify the strengths and limitations of a playground space as it currently stands for practical and financial reasons. Playground audits have been used in research and practice to measure and evaluate attributes of the play space environments (25, 26), and can be employed to evaluate playground inclusion (27). Using auditing tools in existing spaces allows community stakeholders and researchers to evaluate the state of current community playground structures. This process can support the identification of current equipment that meets user needs, and to address limitations and recommend adaptations or modifications to problem areas which better suit the needs of all children (i.e., retrofits, renovations, or re-designs). However, it is unclear if a best-practice tool exists for evaluating the inclusivity of existing playgrounds.

The purpose of this scoping review was to explore available tools for auditing the inclusivity (to enable the participation of children with disabilities) of existing playgrounds in both research and practice settings. The overarching objective was to provide researchers and community stakeholders (e.g., government officials, child development and recreation practitioners, playground developers, and community disability champions) with resources for evidence-based decision-making to improve the inclusivity of playgrounds. The audit process has the potential to engage these diverse groups in meaningful, community-based research through evaluations and advocacy for improved playground environments (26). Unlike previous research which has primarily focused on informing inclusive design for new builds (21–24), this study narrowed the breadth of evidence to examine literature that provides auditing tools to evaluate the design of existing playgrounds, an important and unexplored contribution for community applications.

## 2. Methods

This scoping review was prospectively registered with Open Science Framework (registration #: rycmj) and conforms to the Preferred Reporting Items for Systematic Reviews and Meta-Analysis (PRISMA) extension for Scoping Reviews (28). The protocol for this review was published *a priori* and full study details can be found there (29). The scoping review methodology was selected for this research because it allowed for an appropriate summary of the heterogenous evidence available, with the goal of identifying gaps and informing policy and practice (30, 31).

### 2.1. Search strategy

The search strategy was created and conducted in consultation with a Health Sciences Teaching and Learning Librarian at Western University (Ontario, Canada). In line with the scoping review methodology (31), white and grey literature were included to capture the breadth of auditing tools available in the fields of research and practice. The search was undertaken in a two-phase process.

#### 2.1.1. Phase 1: white literature

The primary search examined empirical, peer-reviewed research (i.e., white literature) which focused on three key themes: 1) the playground environment; 2) children with disabilities (i.e., physical, intellectual, mental, or sensory impairments which interact with barriers to hinder full and equal participation in daily life) (15); and (3), audit tools for evaluating the inclusivity of the playground (refer to the protocol paper for the complete search strategy) (29). Themes 1 and 2 were systematically examined using relevant keywords and medical subject heading (MeSH) terms, combined using Boolean operators and adjusted for four electronic databases: MEDLINE, Scopus, CINAHL, and Embase. Theme 3 was evaluated by hand during screening to account for the variety of terminology used to refer to audit tools. Audit tools were considered broadly as any tool that can be employed to conduct an evaluation of the playground for the inclusion of children with disabilities, using questions that can be completed by a playground auditor (29). Hand searches of the reference lists of included articles and four previous systematic/scoping reviews examining inclusive playground design were undertaken to locate additional eligible white and grey literature (21–24). The final search was conducted on December 18, 2021. All retrieved white literature was exported into the Covidence software for screening and data extraction (32).

To be included, original peer-reviewed research (i.e., white literature) had to be published in English or French since 2000. Studies had to evaluate the inclusivity of existing playground structures (equal access to social and physical aspects of play, regardless of ability) (21, 22), and provide an objective tool (i.e., toolkit, evaluation, audit, checklist, assessment, etc.) to conduct an evaluation of a playground for inclusion of children with disabilities, using questions that can be completed by a playground auditor. Studies were excluded if the full-text article could not be obtained, “playground” was defined in an alternate context (e.g., an environmental playground of bacteria), or the focus of the paper was strictly on the epidemiology of injury or playground safety (21).

#### 2.1.2. Phase 2: grey literature

Using the direction of Godin et al (33)., a grey literature search was conducted by applying a 3-step process for recording the relevant literature. Step 1 involved a search of a grey literature database, the Canadian Health Research Collection Database. Step 2 focused on conducting targeted web-based google searches. Snowball searching was used in Step 3 by examining the reference lists on all white and grey literature included in the full-text screening stages for additional grey literature. The final grey literature search was conducted on March 2, 2022. All retrieved grey literature was exported into Microsoft Excel and assigned a unique identifier for screening and data extraction.

Grey literature (i.e., reports, theses, newspapers, fact sheets, websites, and policy documents produced by government/academics/industry not controlled by commercial publishers) were required to meet al.l inclusion criteria of white literature, with slight modifications (33). Acknowledging the potential volume of grey literature available, an additional inclusion criterion was employed: to ensure that the results of this scoping review reflect best practices for end-users, the grey literature had to report how the tool was developed. Two additional exclusion criteria for grey literature were also applied: 1) secondary applications of tools with unjustified modifications to an original tool reported by another organization; and, 2) examples of organizations applying existing tools in practice. In these situations, the primary source of the tools used was assessed for inclusion in this review.

**Figure 1:**
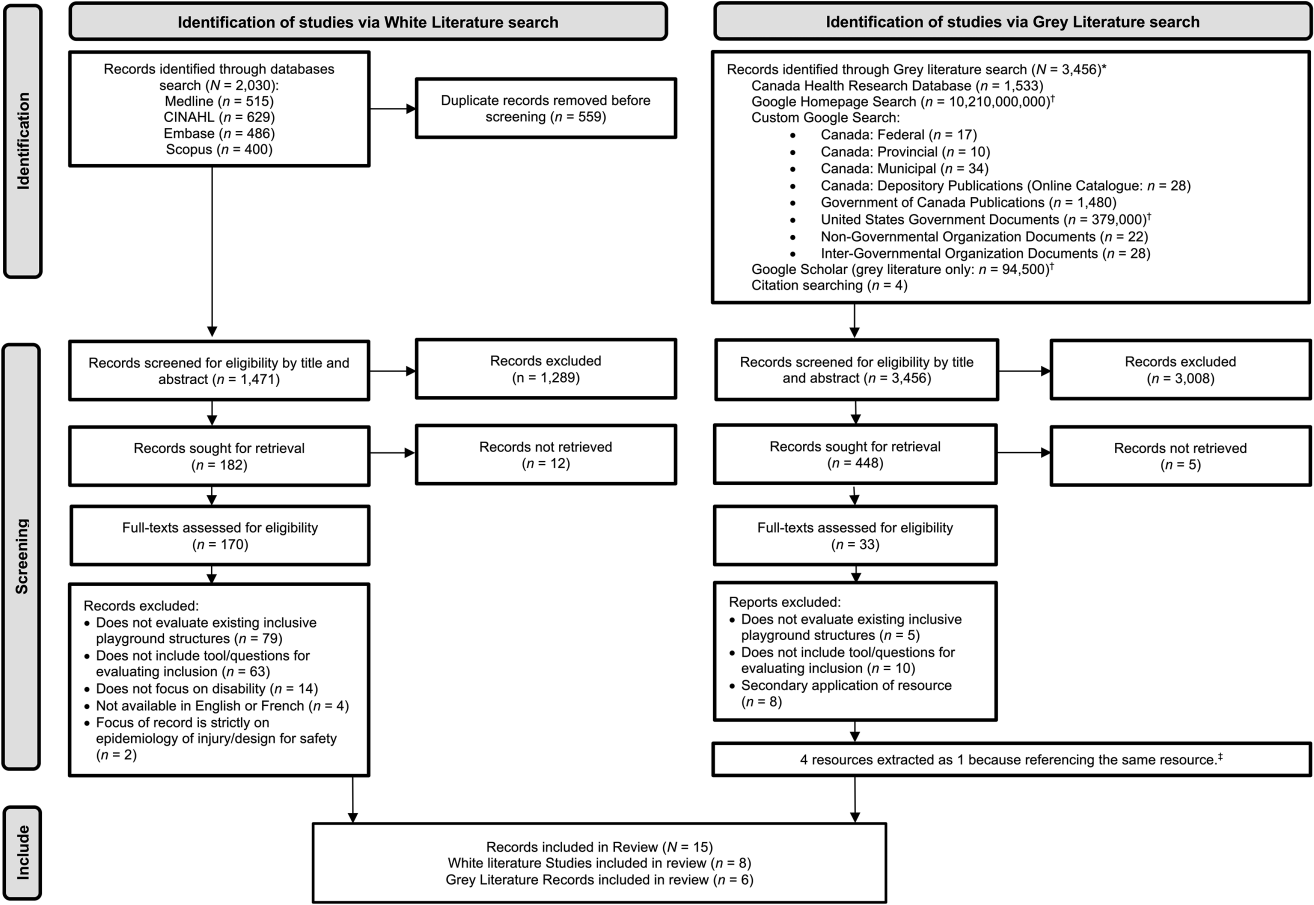
PRISMA flow diagram. *Number indicates search where first 10 pages (i.e., 100 results) were reviewed if results were considered infinite, to capture many of the most relevant hits while still being a feasible amount to screen (Godin et al., 2015). ^†^Searches using first 10 pages of search results only.

The results and inclusion process adhered to, and are presented on, the “PRISMA 2020 flow diagram for new systematic reviews, which included searches of databases, registers and other sources” (see Figure 1) (34).

### 2.2. Screening process & data extraction

Title, abstract, full-text screening, and data extraction of all literature were conducted by two researchers. White literature was managed in Covidence (32), while grey literature was documented in Microsoft Excel. Discrepancies were discussed with a third reviewer until consensus was achieved.

Separate data extraction tables for white (Table 1) and grey (Table 2) literature were generated *a priori*. The tables captured the relevant auditing tool characteristics and detailed all applicable resource information, and results of studies for white literature.

**Table 1:** Characteristics of white literature audit tools.

Tool #*	Author (Year)	Country (province/state/region)	Disability Types Considered	Tool Employed	Environment Assessed	Measure of conceptualization/operationalization	Components Assessed	Grounding Evidence	Tool Strengths	Tool Weaknesses
1	Brennan et al. (2016)	United States (Nebraska)	Not specified	Playground Accessibility Checklist (developed by authors)	2 outdoor playgrounds	Accessibility	Playground features, environmental space demands, playground development/improvement, regulation guidelines, safety	Focus Groups, *ADA Standards*^1^, Occupational Therapy Practice Framework (2014)	Provides a score of overall accessibility on a number of playground components, allowing for comparison between sites. Tool is easily implemented by auditors using typically available materials. Can be complemented by follow up focus groups.	Evaluates accessibility only. Evaluates 8/14 recommendations and promising practice from Brown et al. Psychometric properties not currently assessed. Tool is not conducted with stakeholders present.
2	Lynch et al. (2020)	Ireland (*NA*^2^)	Mental, physical, emotional	PlayAUDIT	5 parks containing playgrounds	Usability, accessibility, playability	Play value and universal design components of play structure	Guided by Centre of Excellence in Universal Design (Ireland)	Evaluates 10/14 recommendations and promising practice from Brown et al., the most of white literature tools. Tool is used to determine play value and universal design components of play structure. Authors followed up with walk and talk audits with playground users. Checklist is easily implemented by multiple auditors using typically available materials.	Psychometric properties not currently assessed. Tool is not conducted with stakeholders present.
3	Olsen & Dieser (2012)	United States (NA)	Physical	ADA Standards Accessibility Checklist	57 parks containing playgrounds	Accessibility	Accessible routes, shade, surface materials, and equipment	ADA Standards	Provides 12 questions to assess accessibility that directly relevant to ADA Guidelines. Checklist is easily implemented by auditors using typically available materials.	Evaluates 5/14 recommendations and promising practice from Brown et al. including only 1/7 features to foster inclusive play. Does not consider user involvement in audit. Psychometric properties not currently assessed.
4	Parker & Al-Maiyah (2022)	England (East Midlands Village)	Mental, physical, emotional	Play Park Evaluation Tool (PPET: III & IV)	20-site pilot study, 1 case study, at parks containing playgrounds	Usability, accessibility, playability	Access, entrances to play park, internal access, non-play equipment, and play equipment	Literature Review, European Safety Standards (EN1176), ADA Guidelines, “BTG-COMP Park observation tool”, ‘Me2 Checklist’ (Playcore), ‘Plan Inclusive Play Checklist’ (InclusivePlay.com)	Measures usability, accessibility, playability. Indicates to be adapted for use by a stakeholders (including children) in the future. Checklist is easily implemented by multiple auditors using typically available materials.	Evaluates 6/14 recommendations and promising practice from Brown et al. Psychometric properties not currently assessed.
5	Perry et al. (2017)	New Zealand (Wellington)	Not specified	PARCs	21 parks containing playgrounds	Accessibility, playability	Surfaces/approachability, play areas and equipment usability/accessibility and play richness, rest areas	Consultation with two disability-focused organizations and officials of three city councils, AIMFREE tool, ADA Standards	Measures accessibility and playability. Checklist is easily implemented by multiple auditors using typically available materials.	Evaluates 7/14 recommendations and promising practice from Brown et al. Does not consider user involvement in audit. Psychometric properties not currently assessed.
6	Rocha et al. (2018)	Brazil (São Paulo)	Physical	Protocol for the evaluation of physical accessibility in schools of Early Childhood Education (Spanish)	4 schoolyard playgrounds at one early years centre	Accessibility	Access to playground, equipment suitability and safety	Brazilian Association of Technical Standards (NBR9050)	Tool is easily implemented by multiple auditors using typically available materials.	Evaluates accessibility only. Evaluates 4/14 recommendations and promising practice from Brown et al. Does not consider user involvement in audit. Psychometric properties not currently assessed.
7	Talay et al. (2010)	Turkey (Ankara)	Physical	Site Surveys	355 outdoor playgrounds	Accessibility	Playground entirely, location, security	Consultation with one disability-focused organization, Convention on the Rights of the Child, the Turkish Constitution and Disability Act	Opinions and observations of parks, including verbal contact with park users are recorded.	Evaluates accessibility only. Evaluates 5/14 recommendations and promising practice from Brown et al. Does not consider user involvement in audit. Psychometric properties not currently assessed.
8	Yantzi et al. (2010)	Canada (Ontario)	Physical	Modified version of the Playability Audit (Ontario Parks Association) compared against ADA Standards	5 schoolyard playgrounds	Accessibility	Access to playground equipment	ADA Standards, ‘Playability Toolkit’ (Ontario Parks Association)	Tool is easily implemented by multiple auditors using typically available materials.	Evaluates accessibility only. Evaluates 3/14 recommendations and promising practice Brown et al. Does not consider user involvement in audit. Psychometric properties not currently assessed.

* Article # corresponds with synthesis of tools in Table 3.

^1^ ADA Standards: Americans with Disabilities Act Standards for Accessible Design.

**Table 2:** Characteristics of Grey Literature Audit Tools.

Tool #*	Organization (Year)	Country (province/state/region)	Disability Types Considered	Environment Assessed	Measure of conceptualization/operationalization	Components Assessed	Grounding Evidence	Tool Strengths	Tool Weaknesses
9	Ontario Parks Association (2001)	Canada (Ontario)	Not specified	Playgrounds in parks	Accessibility, playability	Play space layout, access, amenities	Developed based on consultations with children and adults with disabilities and park industry stakeholders	Measures accessibility and playability. Tool is easily implemented by multiple auditors using typically available materials. Includes playground users with disabilities in audit process.	Evaluates 8/14 recommendations and promising practice from Brown et al. Auditor familiar with relevant policy standards must be present.
10	City of Tempe (2019)	United States (Arizona)	Not specified	Playgrounds in parks	Usability, accessibility, playability	Physical/social/sensory play, access	Created the inclusivity index through adaptation of the Playworld Systems Inclusive Play Design Guide (2013)	Measures usability, accessibility and playability. Provides scoring index for inclusivity index. Tool is easily implemented by multiple auditors using typically available materials.	Evaluates 6/14 recommendations and promising practice from Brown et al. Does not consider user involvement in audit. Majority of the tool focuses on types of play and the equipment that supports this play, instead of general guidelines for accessibility.
11	New South Wales Government (2019)	Australia (New South Wales)	Not specified	Play spaces (accessibility, universal design)	Usability, accessibility, playability	Location, access, play experience, wayfinding, equipment, facilities, landscape	Adapted the Seven Principles of Universal Design (Ron Mace et al., 1997) and the Eight Goals of Universal Design (Steinfield and Maisel, 2012)	Measures usability, accessibility and playability. Tool is easily implemented by multiple auditors using typically available materials. Evaluates 13/14 recommendations and promising practice from Brown et al. Includes playground users with disabilities in audit process.	
12	Inclusive Play Working Group, South Australian Government (2019)	Australia (South Australia)	Not specified	Outdoor play spaces (accessibility, useability)	Usability, accessibility, playability	Environmental barriers/accessibility, amenities, equipment play value	Builds on ‘*Inclusive South Australia’, the State Disability Inclusion Plan 2019-2023*	Measures usability, accessibility and playability. Tool is easily implemented by multiple auditors using typically available materials. Evaluates 13/14 recommendations and promising practice from Brown et al. Includes playground users with disabilities in audit process.	Auditor familiar with relevant policy standards must be present to conduct the playground safety audit.
13	Play Wales, Alison John and Associates (2017)	Wales (Cardiff)	Physical, cognitive, mental, sensory	Play spaces (accessibility, playability)	Accessibility, playability	Key site features, location, care and maintenance, equipment play value, accessibility	Equity Act (2010), focus groups of parents, local authorities responsible for managing play areas, play development officers and children's organizations.	Measures usability, accessibility and playability. Tool is easily implemented by multiple auditors using typically available materials. Evaluates 12/14 recommendations and promising practice from Brown et al. Includes playground users with disabilities in audit process.	This resource would be less intuitive for comparing and contrasting multiple spaces.
14	New England ADA Centre (2010)	United States	Physical	Playgrounds and play spaces (accessibility)	Accessibility	Accessible routes, ground level play components, elevated play components, play components, ground surfaces	2010 Americans with Disabilities Act Standards for Accessible Design	Tool is easily implemented by auditors using typically available materials.	Evaluates accessibility only. Evaluates 7/14 recommendations and promising practice from Brown et al. Tool is not conducted with stakeholders present.

* Article # corresponds with synthesis of tools in Table 3.

### 2.3. Analysis

To compare and synthesize heterogeneous auditing tools, 13 recommendations and one “promising practice” (i.e., area for future research) to design for inclusion by Brown et al. were employed (Table 3) (21). These evidence-based recommendations, developed following a scoping review, provide guidance for designing new playgrounds with consideration to both the physical design and the surrounding built and social environments. The questions from each auditing tool were extracted and synthesized into the recommendations for designing inclusive playgrounds. Frequencies of recommendations employed were calculated and tool applications were explored. Strengths and weaknesses of employing the auditing tools in research or practice were examined (see Tables 1, 2 respectively).

**Table 3:** Synthesis of auditing tool assessments into recommendations and ‘promising practice’ for playground design.

Theme	Recommendations (Promising Practice)	Auditing Tools Providing Relevant Assessments													
		1	2*	3	4*	5*	6	7	8	9	10	11	12	13	14
*Entry Points*	Entrance to the playground space is wide and free of any obstacles	✓	✓	✓	✓			✓		✓		✓	✓	✓	✓
	Wide, flat, and firm pathways from the entrance to the playground	✓	✓	✓		✓	✓	✓		✓	✓	✓	✓	✓	✓
	Enclosing the playground to prevent children from straying (*Promising Practice*)	✓	✓		✓	✓						✓	✓	✓	✓
*Surfacing and paths*	A flat uniform surface that consists of material that is moderately firm and stable	✓	✓	✓	✓		✓	✓	✓	✓	✓	✓	✓	✓	✓
	Ramps that provide access to and between elevated play components	✓	✓	✓		✓	✓	✓	✓		✓	✓	✓		✓
*Features to foster inclusive play*	Play equipment accessible to all children	✓	✓	✓	✓		✓		✓	✓	✓	✓	✓	✓	✓
	Variety of play equipment that provides appropriate challenges for children of all ages and abilities	✓	✓		✓	✓					✓	✓	✓	✓	✓
	Different types of sensory play components that are spread out within the play space to reduce overstimulation		✓		✓	✓					✓	✓	✓	✓	
	Solitary play components for escaping overstimulation					✓				✓		✓	✓	✓	
	Play components shaped in recognizable designs that allow for creative and imaginative pursuits		✓			✓						✓	✓	✓	
	Informational features to aid with spatial orientation, communication, and guidance on proper use of equipment							✓		✓		✓	✓	✓	
	Shaded spaces to aid with temperature regulation									✓		✓	✓	✓	
*Supervision/Staffing*	Trained staff present in the play space to support play for all children														
*Design process*	User involvement (families of children with disabilities and representatives from disability organizations) in the design process	✓	✓							✓		✓	✓	✓	

* Tool is available elsewhere (online, supplementary resource, or via contacting authors.

## 3. Results

The white literature database search yielded 2,030 results with 559 duplicate records. Titles and abstract screening determined 1,289 to be irrelevant; therefore, 167 full-text articles were screened. Eight peer-reviewed studies met the inclusion criteria. The grey literature searches located 3,456 articles. When a google search pulled more than 100 articles (*n* = 4 searches), the first 10 pages of the search results were examined (32). Of these results, 3,008 articles were determined ineligible for inclusion and 448 titles/abstracts were screened. Full-text screening resulted in 10 grey literature articles. Four full-texts were associated with the same article and were extracted as one (17), leaving six grey literature articles. Figure 1 displays a flow diagram of the studies retrieved for the review.

A total of 14 auditing tools were included; published between 2001 and 2022 (half published since 2017), from the United States (*n* = 4), Australia and New Zealand (*n* = 3), the United Kingdom (*n* = 2), Canada, Turkey (*n* = 1), Brazil (*n* = 1) and Ireland (*n* = 1). Specific characteristics of each article including measures of inclusion, disability types considered, tool methodology, and key findings are presented in Table 1 (white literature) and Table 2 (grey literature).

Across tools, inclusion was operationalized using a variety of terminology (i.e., playability, useability, universal design: see Tables 1, 2); however, accessibility specific to the physical space was a consideration in all tools. While these tools were grounded in policy/legislation, stakeholder consultation, and research, none assessed psychometric properties. Disability was often considered strictly in a physical capacity (*n* = 5). Three tools referenced multiple types of disabilities, while six did not specify the disability type being considered (see Tables 1, 2). All tools operationalized disability in relation to the playground environment, focusing on removing barriers to allow for children of all abilities to engage in play.

### 3.1. Descriptive findings

Of the eight peer-reviewed articles (9, 20, 35–40), each used a different tool for evaluating playground accessibility, with five referencing the ADA Standards as their guiding framework (17). All authors emphasized the need for auditing tool development and validation, and for future research to incorporate families who experience disability when establishing research priorities, developing and validating auditing tools, conducting playground assessments, and translating results. See Table 1 for characteristics of white literature.

Among the six tools identified in the grey literature (17, 41–45) all were applications of various policy, legislation or regulation, intended to support practitioners and community stakeholders to upgrade existing playgrounds to be more inclusive for all users. All grey literature expanded beyond the playground/play space to consider the entire park (e.g., bathrooms, parking and access paths). See Table 2 for characteristics of grey literature.

### 3.2. Auditing tools characteristics

When the auditing tools (*n* = 14) were compared to Brown and colleagues' 13 recommendations and one “promising practice” (Table 3) (21), most tools (*n* = 8; 57.1%) provided questions to evaluate more than half of these recommendations. The tools which evaluated less than half of the recommendations were primarily found in the white literature (*n* = 4; 28.6%). Two grey literature tools (both from Australia) provided sufficient information to evaluate against 12 of 13 recommendations (42, 45), and one targeted 10 out of the 13 recommendations (44). All tools evaluated more than 50% of the recommendations in the combined entry points and surfacing/paths categories (accessibility-related categories); however, only six evaluated more than half of the recommendations in the features to foster inclusive play category (9, 38, 42–45), or included user involvement (i.e., families of children with disabilities and representatives from disability organizations) in the design process (9, 35, 42–45). No tools in the literature provided a question to assess supervision/staffing on the playground. See Table 3 for a summary of the questions provided in auditing tools, categorized by the recommendations (and “promising practice”) for playground design from Brown et al. (19).

## 4. Discussion

The purpose of this scoping review was to explore tools available to evaluate the inclusivity of existing playgrounds to enable the participation of children with disabilities. Several evidence-based tools exist to evaluate aspects of inclusion on playgrounds. While accessibility was the main consideration of auditing tools, three grey literature tools aligned well with the recommendations set forth by Brown et al. for designing inclusive playgrounds (19, 42, 44, 45). These tools demonstrate promise for use by researchers, practitioners and community stakeholders (e.g., public health/recreation/government officials, playground developers, and community champions) who are looking to audit the current state of inclusion in their local playgrounds. Several findings warrant discussion.

There were few peer-reviewed articles which employed auditing methodology to evaluate existing playground structures for inclusion, none of which were validated. While no two studies in this review used the same auditing methodology, five referred to the ADA Standards. While this resource provides a focused method for evaluating physical accessibility to playgrounds, it comes at the expense of the wider experience of inclusion (e.g., social accessibility), which is not being captured in playground audits. This presents the potential for a lack of critical engagement in exceeding minimum accessibility standards for play opportunities which include children of all abilities (19).

Evaluating for inclusion, rather than accessibility alone, is key to capturing the experience of children with a wide range of disabilities and providing equitable play opportunities for all. Government legislation and standards can function as a starting point to evaluate accessibility under the umbrella of inclusion (21); however, features to foster inclusive play from usability, playability, and universal design approaches should also be considered. This process should include an examination of the evidence-informed literature captured in this review.

Of the tools examined in this review, one third suggested including families of children with disabilities and representatives from disability organizations in the audit process (9, 35, 42–45). This finding echoes a Dutch study where one quarter of municipalities surveyed had never consulted playground users such as parents and children when designing new community playgrounds (19). A lack of involvement of users in the evaluation and design of playgrounds may mean that barriers to interaction, engagement, and belonging in play may not be adequately caputured (13, 14). If children are not involved in audits, it raises an important question: who is evaluating children's experiences and from what perspective? As emphasized in the literature, it is critical that families with children who have disabilities be engaged in this process as key stakeholders in research and practice to ensure that inclusion is considered and integrated practically. For example, a recent scoping review by Morgenthaler and colleagues suggested that children were knowledgeable about the play value of their community playgrounds, and therefore, their perspectives need to be more closely considered in evaluation of these spaces (46). Not only is it critical to engage playground users in audits to gain insight about playground experiences of inclusion, but also to maximize use of these community spaces by ensuring children's preferences and needs are being met when adaptations are being considered (47).

When undertaking a playground audit to evaluate inclusion, the application of an evidence-based tool can inform decision-making and address relevant priorities. This review identified that although each tool has strengths and limitations, and promising tools exist that will be useful for guiding users in the auditing process, there is currently no best-practice, or widely accepted tool available based on current recommendations for designing inclusive playgrounds (21). The auditing tools identified should be critically appraised prior to use, and considerations such as local users, relevant policy/legislation, and environmental contexts should be considered before use. Applications of these auditing tools would benefit from tailoring tools to local needs based on the gaps identified in this review.

Future development of an auditing tool that allows for consistent, valid assessments of playground inclusion will be important for determining funding allocation, feasibility for upgrading vs. replacing structures, and grassroots advocacy opportunities, to maximize inclusiveness and the overall playground experiences for children with disabilities. Such a tool would have important implications for community-based research, knowledge mobilization, and informing resource allocation (26). Similarities between country-specific playground standards could be drawn on to develop a validated tool that aligns well with all national and international standards. This tool could bridge research and practice to evaluate community settings to better suit the needs of all children.

The present review highlighted gaps in the playground inclusion literature, which should be addressed when considering the future development of an auditing tool. Specifically, it is important that children's health and recreation practitioners, researchers, and government officials establish acceptable standards of inclusion for existing playground structures, which extend beyond accessibility alone. To do this, it is critical to engage with playground users, as they are the ones who play a key role in establishing what a standard of inclusion looks and feels like. Future research should employ qualitative techniques in playground audits to capture these voices. Furthermore, future research should determine best-practice directions which can be used to retrofit existing playgrounds deemed non-inclusive based on the results of audits and investigate how these retrofits affect all children's abilities to engage meaningfully in play. These lines of investigation will help researchers, practitioners, and community stakeholders to better advocate for the inclusion of all children in play opportunities within their communities.

This research makes an important contribution to the literature by systematically summarizing both white and grey literature globally, to provide auditing tools for evaluating the design of existing playgrounds. There are, however, limitations to this review that warrant acknowledgement. First, this study was limited to published white and grey literature available in English and French, introducing the potential for language bias. In two cases, studies published in English provided auditing tools unavailable in English, and therefore, were examined based on the information provided within the article (20, 39). This may have introduced potential bias in the interpretation of the findings. Secondly, for inclusion in this review, the literature had to present an auditing tool to evaluate the inclusivity of existing playground structures. As a result, studies which employed qualitative or survey-based methodology to measure users' perspectives and experiences of inclusion may have been excluded. Finally, while this review sought to systematically examine the grey literature, there are inherent biases involved in this process despite best efforts to ensure scientific rigour (33). Inclusion of the grey literature provided important practical implications for auditing existing playgrounds, and therefore, was undertaken with current best-practice research strategies available for ensuring limited biases of results (33).

Although redesigning and reinstalling new playgrounds that are inclusive for all users, would be ideal, it is not realistic. Based on the results of this review, future applications of the promising tools identified should take into account the local contexts (i.e., users, policy and environment) when conducting audits. Moreover, it is recommended that an auditing tool focusing on inclusion that can be consistently implemented in research and practice settings to evaluate the inclusion of existing playgrounds be developed and validated. This will allow researchers, practitioners and community stakeholders to examine opportunities for improving inclusivity and supporting the health and wellbeing of children with disabilities in their everyday environment through play, a fundamental right of every child.

## Data Availability

The original contributions presented in the study are included in the article/Supplementary Material, further inquiries can be directed to the corresponding author.
